# Early Revision Surgery for Distal Adding-On Correction in Lenke 1 and 2 Adolescent Idiopathic Scoliosis

**DOI:** 10.7759/cureus.30960

**Published:** 2022-11-01

**Authors:** Rami El Rachkidi, Clément Silvestre, Pierre Roussouly

**Affiliations:** 1 Orthopaedics and Traumatology, Hotel-Dieu de France Hospital, Beirut, LBN; 2 Orthopaedic Surgery, Institut de la Colonne Vertébrale (ICV) Lyon Charcot, Lyon, FRA; 3 Orthopaedic Surgery, Centre médico-chirurgical de réadaptation des Massues, Lyon, FRA

**Keywords:** revision surgery, lumbar shift, imbalance, distal adding-on, adolescent idiopathic scoliosis

## Abstract

Objective

To evaluate the outcome of an early revision strategy for postoperative distal adding-on (DAO) after Lenke 1 and 2 adolescent idiopathic scoliosis (AIS) surgery.

Summary of background data

Improper choice of the lowest instrumented vertebra (LIV) is a major cause of postoperative imbalance and unsatisfactory results in AIS surgery. The long-term consequences of such imbalance remain unclear. Early corrective surgery has not been described.

Methods

We retrieved the records of operated AIS patients at the former institution of the senior author. There were 18 cases of early revision by one-level distal extension of instrumentation and fusion. Patients were reoperated based on progressive distal local imbalance and clinical lumbar asymmetry. Several local and global balance parameters were compared on serial long-standing radiographs before and after the index surgery, before and after the revision surgery, and at the last follow-up. The Kruskal-Wallis test was used for the comparison of the results. A value of p<0.05 was considered significant.

Results

All patients were female with a mean age of 13.9 years. The mean delay between the two surgeries was 8.4 months and the last follow-up was at 32.5 months after the revision surgery. Unsatisfactory results after the index surgery were reflected by a progressive increase in disc angulation below the lowest instrumented vertebra (LIV) and an increased tilt and rotation of the LIV+1. The clinical lumbar shift was also accentuated from 19 mm to 25 mm. Revision surgery significantly reduced local and global balance parameters. There was a decrease in the LIV translation (from 26 mm to 19 mm) and of the wedging below it (from 7.9° to 1.3°) and a better positioning of the LIV+1 with less tilt (from 14.6° to 3.6°), translation (from 22.2 mm to 13.8 mm) and rotation (from 20° to 15°). The clinical lumbar shift was reduced from 25 mm to 3.6 mm. Global coronal and sagittal balance were also ameliorated. All results were maintained at a mean follow-up of 32.5 months from the revision surgery. No complications were noted and there was no need for a blood transfusion.

Conclusion

The revision surgery proposed in this paper is simple with low morbidity and may be considered as a fine-tuning of the failed index surgery. Further studies are needed to evaluate the long-term consequences of treated and untreated postoperative distal adding-on in AIS surgery.

## Introduction

Since the development of the Harrington technique [1], surgical management of adolescent idiopathic scoliosis (AIS) continues to evolve. While better correction is the principal corollary of this progress, the goals of surgery for AIS remain the same: prevent the curve's progression, correct the deformity, maintain a balanced spine and preserve as many mobile segments as possible [2]. The latter objective is challenging and competes with the two others. A long fusion offers better correction and a lower risk of postoperative coronal decompensation, progression of the unfused curve, adding-on, and junctional kyphosis but at the price of sacrificing mobile levels [3]. However, for many Lenke 1C, 2C, 3C, and 4C curves where the thoracic curve is the major component of the deformation, spine surgeons believe that a balanced and mobile lumbar spine is much better than a straight and stiff one [4,5]. For this strategy of selective thoracic fusion, the choice of the lowest instrumented vertebra (LIV) is crucial. Recent articles point to the improper choice of the LIV as a major cause of unsatisfactory results [6,7]. While the ideal strategy for choosing the LIV is still debated, surgeons wish to avoid disc wedging and local imbalance below the LIV. The distal adding-on phenomenon is a common complication after AIS surgery and was first reported by Suk et al. [8]. The clinical impact of adding-on is unknown but worsening of the distal lumbar curve can lead to coronal balance decompensation and disappointing results [9]. Distal adding-on often leads to loss of curve correction and unsatisfactory clinical outcomes [10]. In our review of the literature, we could not find any article describing the reintervention technique in cases of early postoperative unsatisfactory results even though revision surgeries for distal adding-on (DAO) were mentioned in some papers [9-13]. At the former institution of the senior author, AIS patients who present with a postoperative progressive distal adding-on, imbalance, and/or lumbar shift were reoperated on an early basis by a simple extension of instrumentation and fusion to the LIV+1 level. This paper describes the reoperation strategy and its results for failed scoliosis surgery in case of an improper LIV.

## Materials and methods

Patients

**Figure 1:**
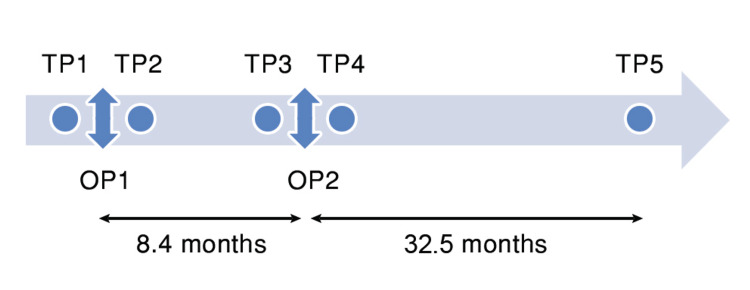
Different time points for radiographic analysis. Before (TP1) and after (TP2) the index surgery (OP1), before (TP3) and after (TP4) the revision surgery (OP2), and at the last follow-up (TP5).

The study received the institution's ethics committee approval. We retrieved the records of AIS patients operated on by the senior author. From a total number of 313 cases, we noted 18 cases of revision for unsatisfactory results by one-level distal extension of instrumentation and fusion. Clinical data and serial standing posteroanterior (PA) and lateral (LAT) radiographs from five different time points (TP) were collected (Figure 1). These included, before (TP1) and after (TP2) the index surgery (OP1), before (TP3) and after (TP4) the revision surgery (OP2), and at the last follow-up (LFU). Radiographs at TP2 and TP4 were generally performed about two weeks postoperatively. The mean delay between OP1 and OP2 was 8.4 months ± 4.5 (range, 3-17 months). LFU was at 32.5 months ± 12 from OP2 (range, 22-65 months). All patients were females with a mean age of 13.9 years (range, 12-15.5 years) at the time of the index surgery. Ten patients had a Lenke 1A curve type; two others had a 1C curve and the remaining 6 others had a 2C curve. Risser sign was 0 in 10 cases, three in five cases, four in two cases, and five in one case. For index surgery, pedicle screws were used exclusively on the concave side and sometimes in association with hooks on the convex side.

Criteria for revision surgery

Criteria for reintervention were distal adding-on, progression of the unfused lumbar curve, local imbalance, junctional kyphosis, and a progressive clinical lumbar shift. Distal adding-on was defined as a progressive increase in the number of vertebrae included within the primary curve distally with an increase of more than 5 mm in the deviation of the LIV+1 from the central sacral vertical line (CSVL) or an increase of more than 5 degrees in the angulation of the first disc below the LIV [14]. The progressive clinical lumbar shift was a common complaint of the patients and their families as it was responsible for a clear asymmetry of the lumbar folds. 

Important steps in the revision surgery technique 

**Figure 2:**
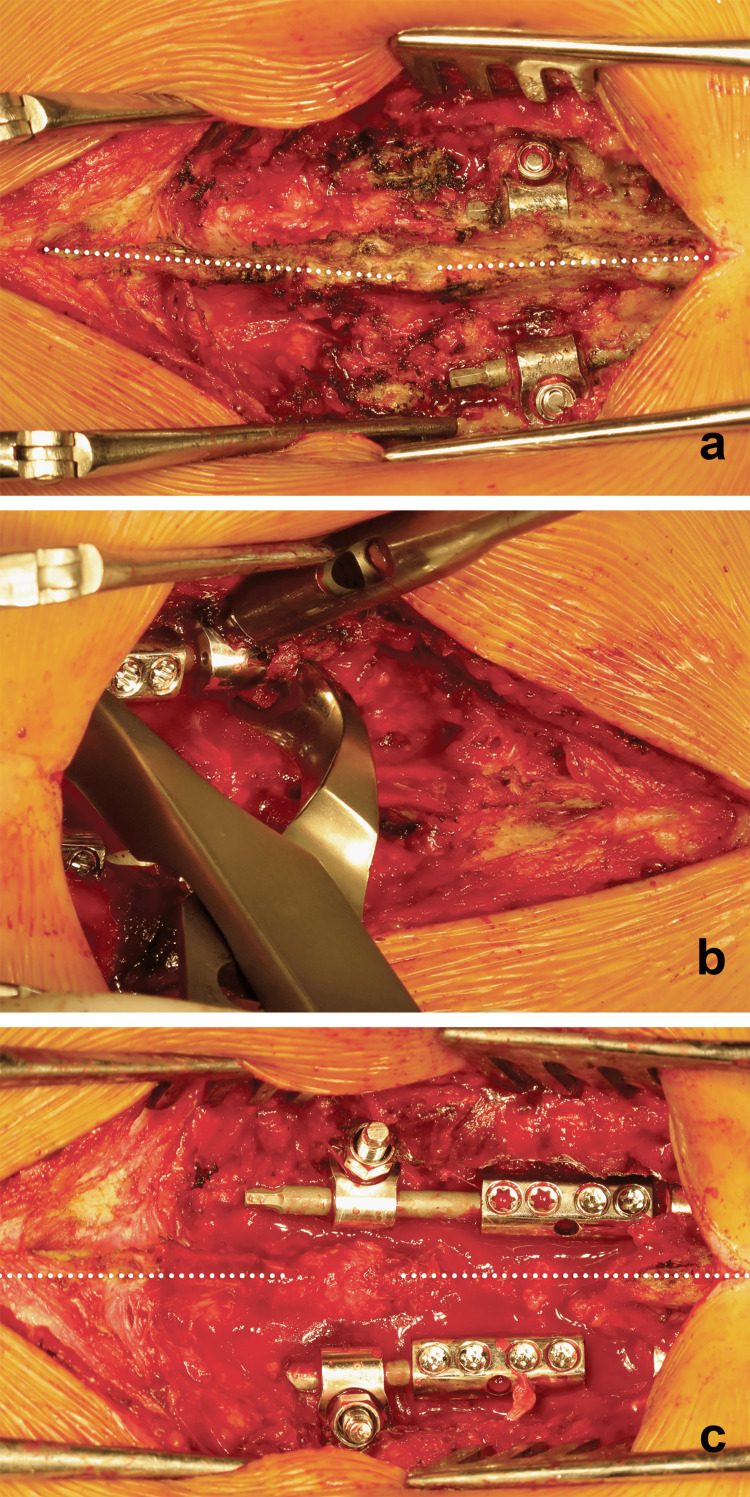
Steps in revision surgery a) Exposure of the LIV, the LIV+1, and the distal instrumentation showing the negative effect of disc wedging on spine alignment (dotted lines). b) Applying compression on the side of the disc opening after removal of the distal pair of screws, placement of pedicle screws in the LIV+1, and extension of the instrumentation using rod connectors. c) Final construct with proper alignment of fused and unfused segments.

The skin incision is centered over the distal end of the previous index surgery scar to expose the LIV, the distal instrumentation, and the level just below the LIV (LIV+1 level). The connections of the LIV screws to the rods were removed. Removal of the distal pair of screws is mandatory to connect the previous instrumentation to the newly inserted screws of the LIV+1. Compression and distraction are made according to the deformity. A preoperative radiograph confirms the good positioning of the LIV+1 which becomes the new LIV. The three main steps of the revision surgery are shown in Figure 2. The mean duration of surgery was 54.5 minutes ± 10.1 (range, 35-65 minutes). Bleeding was evaluated to 222 ml ± 116 ml (range, 75-450 ml). Hospital stay length was 2.7 days (range, 2-5 days). No complications were noted and no blood transfusion was needed in any case.

Radiographic measurements

**Figure 3:**
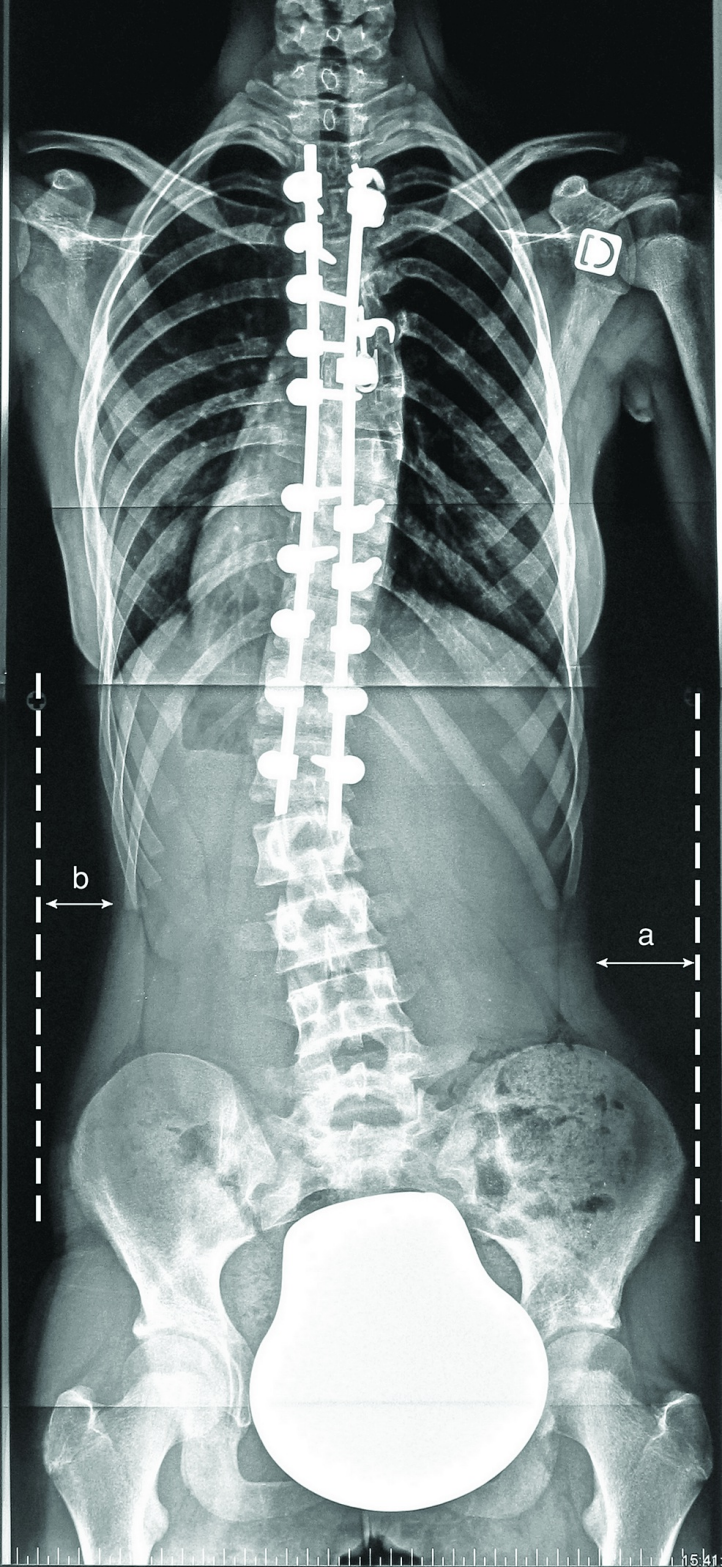
Measuring the clinical lumbar shift Difference between the maximal translation of lumbar soft tissues on both sides from the vertical lines to the soft tissues covering the iliac wings (a-b). A negative value means a shift to the right side.

One spine surgeon, not involved in the performed surgeries, did all the radiographic measurements. Coronal measurements included thoracic and lumbar curves cobb angles, translation of the C7 plumbline from the central sacral vertical line (CSVA) for global coronal balance evaluation, the LIV/LIV+1 cobb angle, the tilt, the translation and the rotation (Nash-Moe method) of the LIV and the LIV+1 vertebras and the disc angulation (wedging) below these vertebras. As the clinical lumbar soft tissues’ asymmetry was a major postoperative complaint, we chose to measure it using digital radiographs by changing the image contrast: we calculated the difference between maximal translations of lumbar soft tissues on both sides from the vertical lines to the soft tissues covering the iliac wings (Figure 3).

Lateral radiographs measurements included the maximal thoracic kyphosis, the maximal lumbar lordosis, the displacement of the C7 plumbline relative to the posterior-superior corner of S1 (PSCS), the angle between the LIV and LIV+2 vertebras for junctional kyphosis evaluation. Pelvic incidence, sacral slope, and pelvic tilt were also measured. A negative sign was arbitrarily attributed to a coronal C7 plumbline and a lumbar shift to the right of the CSVL, a disc opening to the right, a vertebral tilt to the left, a vertebral translation to the right of the CSVL, a kyphosis for LIV/LIV+2 sagittal angle and a sagittal C7 plumbline falling behind the PSCS.

Statistical analysis

The comparison of each radiographic parameter at different time points was performed using the Kruskal-Wallis test, followed by Conover Iman for multiple pairwise comparisons. Differences were considered significant for a value of p<0.05.

## Results

Preoperative data

Preoperative data are shown in Table 1. The mean thoracic and lumbar Cobb angles were 52° and 46° respectively. All cases were globally balanced with a mean value of 9.2 mm and extreme values not deviating more than 20 mm from the CSVL. The LIV/LIV+1 Cobb angle was 13.3° with a lumbar trunk shift of 16 mm (range, 4-46 mm). The tilt, the translation, and the rotation of the LIV were 10.8°, 24.4 mm, and 18° respectively while the disc angulation (wedging) just below, was 7.1°. Corresponding values for the LIV+1 were 15.8°, 20.2 mm, 15° and 4.5°. Sagittal radiographic parameters showed a mean thoracic kyphosis of 30° and a mean lumbar lordosis of 56°. The mean lordosis angle at the LIV/LIV+2 levels was 20° (lowest value = 2°). The mean position of the sagittal C7 plumbline was in front of the PSCS by 16.4 mm. Pelvic incidence had a mean value of 60° and a pelvic tilt of 19°. 

**Table 1:** Mean values and ranges of preoperative parameters MT: main thoracic curve, TL/L: thoracolumbar/lumbar curve, LIV: lowest instrumented vertebra, LIV+1: first vertebra below the LIV, LIV+2: second vertebra below the LIV, SD: standard deviation, Min: minimum, Max: maximum

Parameters	Mean ± SD	Ranges (Min/Max)
Age (years)	13.9 ± 1.4	12/15.5
Weight (kg)	49.4 ± 9	35/60
Height (cm)	164 ± 6.1	155/171
Risser sign	2 ± 2.1	0/5
MT Cobb angle (°)	51.8 ± 13.6	36/71
TL/L Cobb angle (°)	46.4 ± 17.1	20/70
Global coronal balance (mm)	9.2 ± 7.3	-18/20
LIV/LIV+1 Cobb angle (°)	13.3 ± 7.3	2/26
Lumbar trunk shift (mm)	16 ± 12.7	4/46
Wedging below the LIV (°)	7.1 ± 2.9	3/13
LIV tilt (°)	10.8 ± 7.7	-4/22
LIV translation (mm)	24.4 ± 11.6	-20/44
LIV rotation (%)	18 ± 11	0/35
Wedging below the LIV+1 (°)	4.5 ± 1.9	-3/7
LIV+1 tilt (°)	15.8 ± 6.9	8/30
LIV+1 translation (mm)	20.2 ± 12.4	-6/36
LIV+1 rotation (%)	15 ± 7	6/25
Maximal thoracic kyphosis (°)	30.3 ± 18.1	8/62
Maximal lumbar lordosis (°)	56.6 ± 12.7	40/78
LIV/LIV+2 lordosis (°)	20.2 ± 13.9	2/42
Global sagittal balance (mm)	16.4 ± 23.5	-20/40
Pelvic incidence	60.1 ± 13.2	40/72
Sacral slope	41 ± 13	10/54
Pelvic Tilt	19.1 ± 14.6	4/54
Fusion T3-L2	6	
Fusion T3-L1	8	
Fusion T2-L1	3	
Fusion T5-L2	1	

Postoperative data

Postoperative data are summarized in Table 2.

**Table 2:** Mean radiographic measurements at the five-time points (TP) MT: main thoracic curve, TL/L: thoracolumbar/lumbar curve, LIV: lowest instrumented vertebra, LIV+1: first vertebra below the LIV, LIV+2: second vertebra below the LIV. NS: Not significant. Standard deviation values are not shown.

Parameters	TP1	p (TP1-TP2)	TP2	p (TP2-TP3)	TP3	p (TP3-TP4)	TP4	p (TP4-TP5)	TP5
MT Cobb angle (°)	51.8	<0.001	18.8	NS	20.5	NS	19	NS	19.4
TL/L Cobb angle (°)	46.4	<0.001	23.3	NS	25.7	0.005	19.9	NS	18
Global coronal balance (mm)	9.2	NS	7	NS	10.2	0.044	4.1	NS	3.1
LIV/LIV+1 Cobb angle (°)	13.3	NS	11.1	0.011	14.8	<0.001	6.5	NS	4.6
Lumbar shift (mm)	16	NS	19.2	0.038	24.9	<0.001	3.6	NS	3.8
Wedging below the LIV (°)	7.1	NS	5.2	0.046	7.9	<0.001	1.3	NS	1.5
LIV tilt (°)	10.8	0.015	4.1	NS	3.9	NS	3.5	NS	4.4
LIV translation (mm)	24.4	NS	21.2	NS	25.8	0.006	15.2	NS	12.8
LIV rotation (%)	18	NS	18	NS	22	NS	17	NS	15
Wedging below the LIV+1 (°)	4.5	NS	3.1	NS	3.8	NS	2.5	NS	3.8
LIV+1 tilt (°)	15.8	0.04	9.9	0.004	14.6	<0.001	3.6	NS	2
LIV+1 translation (mm)	20.2	NS	18.8	NS	22.2	0.009	13.8	NS	11.8
LIV+1 rotation (%)	15	NS	15	0.047	20	0.007	15	NS	15
Maximal thoracic kyphosis (°)	30.3	NS	32	NS	31.7	NS	30.9	NS	32.8
Maximal lumbar lordosis (°)	56.6	NS	54.4	NS	57.5	NS	61.9	NS	60.9
LIV/LIV+2 lordosis (°)	20.2	NS	19.2	NS	19.6	NS	20.1	NS	19.3
Global sagittal balance (mm)	16.4	NS	19.8	NS	8.2	0.035	-4.4	NS	3.1
Sacral slope	41	NS	44	NS	45	NS	43	NS	45
Pelvic Tilt	19.1	NS	16	NS	15	NS	17	NS	15

**Figure 4:**
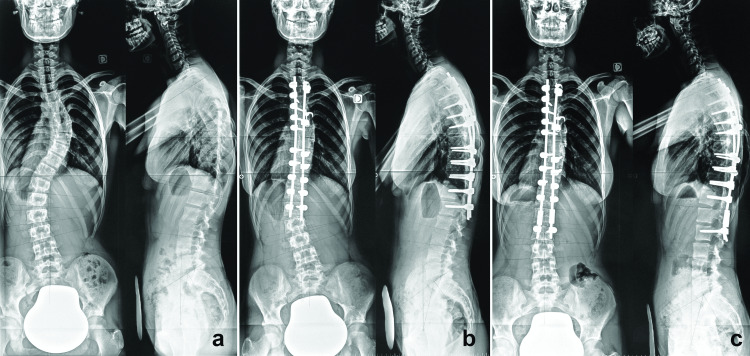
A representative case of the series a) Preoperative postero-anterior and lateral radiographs (Lenke 1C curve). b) Radiographs six months after index surgery (OP1) with a clear global and local imbalance from excessive disc wedging below the LIV. A distal junctional kyphosis is noted. c) Radiographs at 26 months from revision surgery showing constancy of the amelioration.

Measurements at TP2 showed a significant correction of both thoracic and lumbar curves which is the primary objective of the surgery. This correction brought the LIV and the LIV+1 to a more horizontal position reducing their tilt. Although statistically insignificant, we noticed a tendency towards the aggravation of clinical lumbar shift and global coronal balance (from 16 mm to 19.2 mm and from 7 mm to 10.2 mm respectively). No variations were noted postoperatively in all sagittal parameters. In the 8.5-month postoperative period (TP2-TP3), there was an increase in local imbalance due to an excessive wedging below the LIV (from 5.2° to 7.9°) with subsequent increase in the LIV/LIV+1 Cobb angle (from 11.1° to 14.8°) and in the lumbar shift (from 19.2 mm to 24.9mm). Because of this wedging, the LIV+1 tilt reincreased to near preoperative values. A higher rotation of the LIV+1 was another aspect of this unsatisfactory result. By extending the instrumentation to the LIV+1 level (TP4), major modifications occurred with the correction of all local parameters around the distal instrumentation leading to a better global coronal, sagittal and local balance. The improvement in radiographic parameters had a clear repercussion on the clinical aspect by decreasing the lumbar shift from 24.9 mm to 3.6 mm. All results were maintained at the last follow-up (TP5). A typical case is shown in Figure 4.

## Discussion

Choosing the “correct” LIV is one of the most challenging aspects of AIS surgery. By “correct”, we mean the LIV that can save as many mobile lumbar levels as possible while respecting the goals of the surgery. This problem is far from being completely solved because of the three-dimensional complexity of the deformity and bad outcomes are still reported. Postoperative decompensation after selective thoracic fusion for thoracic AIS has been attributed to improper fusion levels [7]. Failure to appropriately select the LIV can lead to fusions that are either too long with less mobile levels or too short resulting in negative outcomes such as decompensation [8]. we believe that it is hard to find a generally applicable rule for all types of curves and that every case needs proper preoperative planning. In case of unsatisfactory postoperative clinical and radiographical results, surgeons may accept the suboptimal outcome to avoid a complex scoliosis revision surgery. The relatively simple and safe revision surgery described in this paper was thoroughly discussed with the family after noticing the progressive adding-on phenomenon. Patients and their parents were not satisfied with the initial result and accepted the second surgery, considered as a fine-tuning of the first one.

Coronal imbalance is usually defined as a translation of more than 20 mm of the C7 plumbline from the CSVA [8]. Global coronal balance appears to stabilize three months after posterior vertebral fusion for AIS and one should note hope for imbalance correction after this period [15]. In our series, we found that global imbalance rarely exceeded 20 mm and was not a reason for reintervention. The improper choice of LIV had repercussions on the local balance around the distal end of instrumentation. Global imbalance is compensated at the expense of balance in the distal area of the fused segment. A “bad” result may be suspected in the early postoperative period with wedging below the LIV, increased tilt or translation of the LIV, adding-on, or local imbalance. Zhao et al. [16] found that lateral disc opening, two weeks postoperatively, is the best predictor of the two-year postoperative disc opening. However, local imbalance may not be progressive and in many cases, serial radiographs show improvement in radiographic parameters over a few months. In some other cases, worsening occurs with a progressive increase of LIV wedging and of the local Cobb angle. The increasing tilt and rotation of the LIV + 1 vertebra found in our series are consistent with the progression of the deformity below the LIV. Patients were never reoperated before three months, giving them the chance for spontaneous correction. The main reason for reoperation is the progressive and continuous deterioration of the radiographic parameters and of the clinical aspect. In all cases of our series, serial radiographs never showed improvement. Once the progressive deterioration is confirmed after three months of follow-up, reoperation was planned after discussion with the family. As the second surgery is intended in part to minimize the potential degenerative changes secondary to improper balance [17], there was no reason to postpone the surgery for a long time. In all cases, we took the decision in the first year with a mean of 8.4 months. Painful conditions of patients with progressive DAO gradually worsen during follow-up [18].

Wedging and translation are the two major components of local imbalance. Distal adding-on is often accompanied by an unsatisfactory clinical outcome and a high risk of reoperation [10,9]. This problem was demonstrated in our series with a significant progression of disc wedging from 5° to 8° in an 8.5-month period (p=0.04). OP2 directly corrected the wedging down to 1.3° (p<0.001) and maintained the results at a mean follow-up of 32.5 months. Another effect of OP2 is the decrease in LIV and LIV+1 translations, accounting for an additional correction of balance. 

Despite the small decrease in mean LIV/LIV+2 lordosis, a 10° loss of lordosis was noted in two of our cases in only five months postoperatively. Fusion to the LIV+1 level led to little improvement in lumbar and LIV/LIV+2 mean lordosis, but this was sufficient to reach initial preoperative values and notably improved the sagittal balance. Since it is desirable to keep the LIV and subjacent unfused disc horizontal [16], we believe that the better positioning of the “new” LIV, with less rotation, translation and tilt had a protective effect on the lower unfused levels, maintaining a good balance at the two-years the last follow-up. 

**Figure 5:**
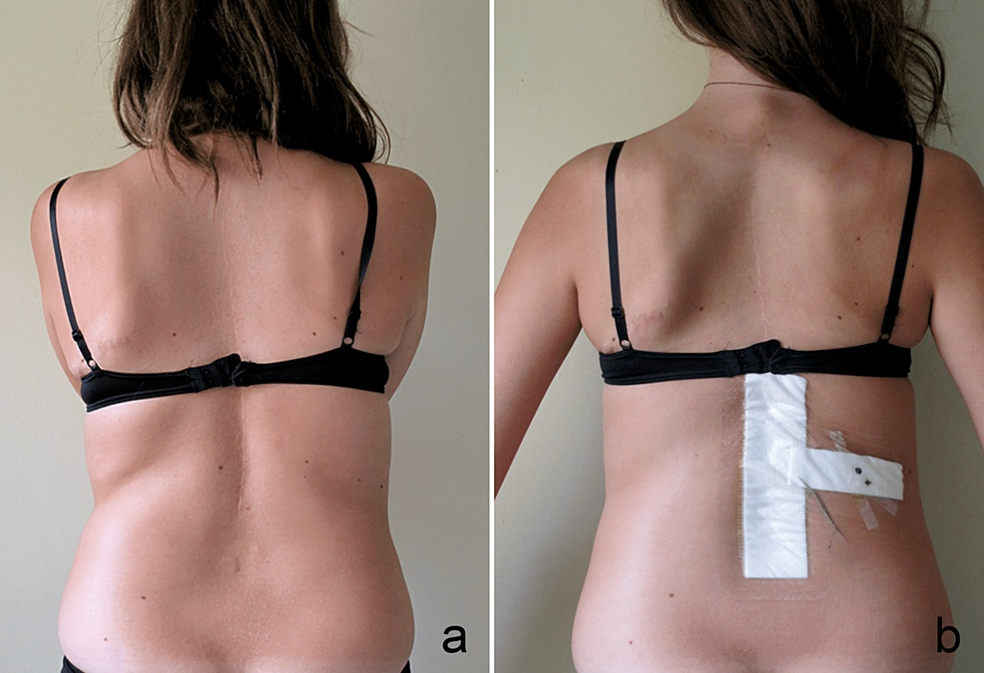
Clinical lumbar shift before (a) and after (b) the revision surgery

Another important feature of the initial failed surgery is the progressive lumbar deviation due to local imbalance. A patient may be “balanced” in the coronal plane, with the head centered over the pelvis, but may still show a significant trunk imbalance. Trobisch et al. [19] found that iatrogenic postoperative trunk shift has an incidence of 8.8% in the surgical treatment of AIS. They studied the position of the rib cage in reference to the central sacral vertical line (CSVL). We think that our method of evaluating the trunk shift is more accurate as it better reflects the clinical aspect. In our series, increased lumbar asymmetry was a major complaint of young girls and their parents (Figure 5).

Some patients had an obvious lumbar folds’ asymmetry while having a C7 plumbline that does not deviate from the CSVL. Four patients had a lumbar shift of 4 cm before the second intervention which led to an unpleasant clinical asymmetry in the lumbar region. Reoperation decreased considerably the lumbar asymmetry and improved the clinical aspect. Unlike the uncertainty about lowering the risk of secondary degenerative changes decades later, the revision surgery offers an immediate correction of the lumbar asymmetry. 

The revision surgery described in this paper is technically simple with low morbidity and may also be applied to add more than one level to the distal fusion. We believe that it lowers the risk of degenerative changes at an older age requiring more complex surgery. We had two cases reoperated for a two-level distal extension of instrumentation with similar results, but they were not integrated into this series to keep it homogeneous for radiographic measurements.

Our study has many limitations. Firstly, it is a retrospective study without a control group. Secondly, there are only 18 cases, making it difficult the formulation of clear guidelines for revision surgery. Finally, we didn’t use quality-of-life questionnaires because many patients didn’t have scores collected preoperatively.

## Conclusions

The revision surgery proposed in this paper is simple and may be considered as a fine-tuning of the failed index surgery. It brings an immediate correction to the imbalance and its corollary, the disagreeable lumbar folds asymmetry. On the other hand, this surgery is intended to avoid future potential degenerative changes distal to the fused segment many years following the initial surgery. We strongly believe that the benefits of an early revision outweigh the risks of this additional surgery; however, further studies are needed to evaluate the long-term consequences of treated and untreated postoperative local imbalance.

## References

[1] Harrington PR (1962). Treatment of scoliosis. Correction and internal fixation by spine instrumentation. J Bone Joint Surg Am.

[2] Fischer CR, Kim Y (2011). Selective fusion for adolescent idiopathic scoliosis: a review of current operative strategy. Eur Spine J.

[3] Yang C, Li Y, Yang M, Zhao Y, Zhu X, Li M, Liu G (2016). Adding-on phenomenon after surgery in Lenke type 1, 2 adolescent idiopathic scoliosis: is it predictable?. Spine (Phila Pa 1976).

[4] Jiang H, Shao W, Xu E (2018). Coronal imbalance after selective posterior thoracic fusion in patients with Lenke 1 and 2 adolescent idiopathic scoliosis. Biomed Res Int.

[5] Ohrt-Nissen S, Luk KD, Samartzis D, Cheung JP (2020). Selection of the lowest instrumented vertebra in main thoracic adolescent idiopathic scoliosis: Is it safe to fuse shorter than the last touched vertebra?. Eur Spine J.

[6] Kwan KY, Wong CP, Koh HY, Cheung KM (2019). Selection of lowest instrumented vertebra using fulcrum bending radiographs achieved shorter fusion safely compared with the last "substantially" touching vertebra in Lenke Type 1A and 2A curves. Spine (Phila Pa 1976).

[7] Yin R, Qin X, He Z, Liu Z, Qiu Y, Zhu Z (2021). Which thoracic curves are at the greater risk for distal adding-on: comparison between typical and atypical Lenke 1A curves. Eur Spine J.

[8] Suk S-I, Lee S-M, Chung E-R, Kim J-H, Kim W-J, Sohn H-M (2003). Determination of distal fusion level with segmental pedicle screw fixation in single thoracic idiopathic scoliosis. Spine (Phila Pa 1976).

[9] Lakhal W, Loret JE, de Bodman C, Fournier J, Bergerault F, de Courtivron B, Bonnard C (2014). The progression of lumbar curves in adolescent Lenke 1 scoliosis and the distal adding-on phenomenon. Orthop Traumatol Surg Res.

[10] Cao K, Watanabe K, Kawakami N (2014). Selection of lower instrumented vertebra in treating Lenke type 2A adolescent idiopathic scoliosis. Spine (Phila Pa 1976).

[11] Sponseller PD, Betz R, Newton PO (2009). Differences in curve behavior after fusion in adolescent idiopathic scoliosis patients with open triradiate cartilages. Spine (Phila Pa 1976).

[12] Schulz J, Asghar J, Bastrom T (2014). Optimal radiographical criteria after selective thoracic fusion for patients with adolescent idiopathic scoliosis with a C lumbar modifier: does adherence to current guidelines predict success?. Spine (Phila Pa 1976).

[13] Upasani VV, Hedequist DJ, Hresko MT, Karlin LI, Emans JB, Glotzbecker MP (2015). Spinal deformity progression after posterior segmental instrumentation and fusion for idiopathic scoliosis. J Child Orthop.

[14] Wang Y, Hansen ES, Høy K, Wu C, Bünger CE (2011). Distal adding-on phenomenon in Lenke 1A scoliosis: risk factor identification and treatment strategy comparison. Spine (Phila Pa 1976).

[15] Leroux J, Lechevallier J, Griffet J (2011). Abstracts of the SFCR congress 2011 (Frenc Spine Society). Eur Spine J.

[16] Zhao Y, Wang Z, Zhu X, Wang C, He S, Li M (2011). Prediction of postoperative trunk imbalance after posterior spinal fusion with pedicle screw fixation for adolescent idiopathic scoliosis. J Pediatr Orthop B.

[17] Meir AR, Fairbank JC, Jones DA, McNally DS, Urban JP (2007). High pressures and asymmetrical stresses in the scoliotic disc in the absence of muscle loading. Scoliosis.

[18] Qin X, Xia C, Xu L, Sheng F, Yan H, Qiu Y, Zhu Z (2018). Natural history of postoperative adding-on in adolescent idiopathic scoliosis: what are the risk factors for progressive adding-on?. Biomed Res Int.

[19] Trobisch PD, Samdani AF, Pahys JM, Cahill PJ (2011). Postoperative trunk shift in Lenke 1 and 2 curves: how common is it? and analysis of risk factors. Eur Spine J.

